# Evidence that growth hormone can improve mitochondrial function in oocytes from aged mice

**DOI:** 10.1530/REP-18-0529

**Published:** 2019-01-21

**Authors:** Hai-Yan Hou, Xi Wang, Qi Yu, Hong-Yi Li, Shao-Jie Li, Rui-Yi Tang, Zai-Xin Guo, Ya-Qiong Chen, Chun-Xiu Hu, Zhi-Juan Yang, Wen-ke Zhang, Yan Qin

**Affiliations:** 1 Chinese Academy of Medical Sciences & Peking Union Medical College, Peking Union Medical College Hospital, Beijing, People’s Republic of China; 2 Department of Obstetrics and Gynecology, Characteristic Medical Center of PAP, Tianjin, People’s Republic of China

## Abstract

Decline in successful conception decreases more rapidly after 38 years of age owing to follicular depletion and decreased oocyte quality. However, limited information is available regarding the underlying mechanism and the useful treatment. This study aimed to evaluate the effects of growth hormone supplementation on oocyte maturation *in vivo* in aged and young mice and to determine its effect on mitochondrial function. The influence of three different doses of recombinant human growth hormone (rhGH) (0.4, 0.8 and 1.6 mg/kg/day) for 8 weeks before ovarian stimulation was analyzed. Superovulated oocytes were released from the oviduct of 12-week-old and 40-week-old female C57BL/6J mice 14–16 h after administration of human chorionic gonadotropin. Ovarian follicle and morphological analysis and oocyte maturation parameters were then evaluated. This study is the first, to our knowledge, to report that medium- and high-dose rhGH significantly increases antral follicles in aged mice but anti-Müllerian hormone (AMH) levels. Furthermore, derived oocytes, MII-stage oocyte rate, ATP levels, mitochondrial membrane potential and frequencies of homogeneous mitochondrial distribution increased. In contrast, in both aged and young mice, the mtDNA copy numbers per oocyte were similar before rhGH administration, and upon saline administration, they did not differ significantly. We conclude that medium-dose rhGH supplementation before standard ovarian stimulation regimens improves oocyte quality in aged mice, probably by enhancing mitochondrial functionality.

## Introduction

The probability of successful conception dramatically declines nonlinearly with maternal age in a nonlinear manner. Decline in successful conception decreases more rapidly after 38 years of age (Faddy 2000) owing to the progressive decline in ovarian follicles and decreased oocyte quality (Perheentupa & Huhtaniemi 2009). Adequate patient response to ovarian stimulation determines the number and quality of oocytes available for, and the successful outcome of, *in vitro* fertilization (IVF). Clinical studies have reported that women over 40 years of age undergoing IVF with donated oocytes have pregnancy rates comparable to those of young patients. The consistent live birth rate, irrespective of maternal age, suggests that decline in oocyte quality majorly contributes to age-related infertility. Ovarian aging is associated with defects in chromatid separation and chromosome decondensation (Tarin *et al.* 2001) and spindle detachment causing chromosomal misalignment (Liu & Keefe 2002). However, limited information is available regarding the precise cause and mechanism underlying the decline in oocyte quality, and only a few targets have been identified. To date, no treatment method has successfully improved the chances of a live birth in women of advanced maternal age.

Several potential molecular mechanisms contribute to ovarian aging, including metabolic/energetic disorders, telomere shortening (Kalmbach *et al.* 2015), impaired DNA repair (Titus *et al.* 2015) and mitochondrial dysfunction (Wang *et al.* 2017). Mitochondria are the cellular energy producers and are the most abundant organelles within oocytes. Emerging evidence has demonstrated the role of mitochondria in oocyte development and reproduction. Oocyte maturation is complex and involves nuclear, cytoplasmic and epigenetic changes that culminate in the formation of the meiotic spindle. All these processes require energy, which is provided by the mitochondria via the oxidative phosphorylation (OXPHOS) system (Dumollard *et al.* 2007). OXPHOS is a metabolic pathway where in cells use enzymes to oxidize nutrients, release energy and produce ATP. Presently, the most critical factor in oocyte fertilization and embryo developmental competence is the mitochondria’s ability to maintain ATP homeostasis. Higher ATP levels in oocytes correlate with improved embryonic development and implantation rates (Van Blerkom *et al.* 1995). OXPHOS insufficiencies and/or deterrents to mitochondrial function may yield reactive oxygen species (ROS), potentially causing cellular dysfunction including the arrest of oocyte maturation, chromosomal misalignment and compromised embryo development and/or apoptosis (Liu & Keefe 2002, Takeuchi *et al.* 2005, Thouas *et al.* 2005, May-Panloup *et al.* 2016). Oocyte aneuploidy rates increase with age, and it is believed that increased aneuploidy is believed to be the mechanism responsible for decreased oocyte quality (Hassold & Chiu 1985, Hunt & Hassold 2008, Selesniemi *et al.* 2011, Duncan & Gerton 2018, Severance & Latham 2018).

Mitochondria are unique organelles as they contain DNA (mtDNA). mtDNA copy number and the timing of mtDNA replication during oocyte maturation determines the fertilization outcome of oocytes (El *et al.* 2006, Viet *et al.* 2013, Fragouli & Wells 2015, May-Panloup *et al.* 2016). mtDNA copy number is lower in unfertilized oocytes of women over 40 years of age, indicating defective cytoplasmic maturation (Murakoshi *et al.* 2013). Studies on murine controlled ovarian hyperstimulation (COH) have reported that reductions in mitochondrial copy number, membrane potential and ATP content in oocytes correlate with reductions in mtDNA copy number which is a potential biomarker of embryo viability.

Except for oocyte donation, there is no known intervention to improve the pregnancy outcomes in older patients. However, improving mitochondrial function using nutrients or procedures involving mitochondrial transfer can enhance fertility outcomes. Mitochondrial nutrients are biological or chemical compounds that boost the mitochondrial energy-producing capacity. Recent studies have harnessed the association between oocyte quality and mitochondrial nutrients including α-lipoic acid (Tibullo *et al.* 2017), coenzyme Q10 (Ben-Meir *et al.* 2015), resveratrol (Liu *et al.* 2018) and recombinant human growth hormone (rhGH) (Weall *et al.* 2015). rhGH has recently gained increasing attention as an anti-aging compound increasing functional mitochondrial numbers in aged women with poor ovarian responses when applied during COH cycles. Co-stimulation with rhGH improved mitochondrial function and yielded better outcomes including increased numbers of oocytes collected and embryos obtained (Yi & Maeda 2005, Lagouge *et al.* 2006, Pearson *et al.* 2008). However, few studies provide direct evidence regarding mitochondrial dysfunction in oocyte quality in aging oocytes. Furthermore, no studies have attempted to identify the potential benefits of rhGH in young female mice. It is still unclear whether rhGH improved oocyte competence in aged and young animals as is its optimal dose. In the present study, we assessed the effects of rhGH on *in vivo* aged and young ovulated oocytes using mouse models. Understanding the potential effects and mechanisms of rhGH-mediated improvement of oocyte quality in aging females would have significant medical and social implications.

## Materials and methods

### Animal and experimental design

Female C57BL/6J mice, at 4 (~20 g) and 32 weeks of age (~30 g), were obtained from Beijing HFK Bioscience Co. Inc. Both young (4–5 weeks) and aged (32–33 weeks) mice were assigned randomly to experimental and control groups and treated for 8 weeks. The treatment groups comprised (i) an untreated control group (wild-type (WT)); (ii) a negative control group of mice receiving a saline injection once daily (150 µL and 200 µL for young and aged mice, respectively); three experimental groups, injected once daily with 150 µL or 200 µL of either; (iii) 0.4 mg/kg (low-dose); (iv) 0.8 mg/kg (medium-dose) or (v) 1.6 mg/kg (high-dose) recombinant human growth hormone (rhGH, Jintropin, GenLei, China), dissolved in saline. Mice were housed in a temperature-controlled environment (22–24°C) under regulated light (12:12-h light/darkness cycle) in the animal facility and fed a standard pellet diet with free access to water. The present study was performed with ethical approval from the Committee of Animal Experimentation of the Chinese People’s Armed Police Force Logistic College Hospital. Animals were treated in accordance with the National Institutes of Health’s Guide for the Care and Use of Laboratory Animals.

### Estimation of anti-Müllerian hormone (AMH) levels

Mice from each group were killed via cervical dislocation under anesthesia before collecting blood samples. Serum was separated via centrifugation at 3000 ***g*** for 15 min 4°C and stored at −80°C until assayed. AMH levels were assessed using a sensitive electrochemical luminescence method (Elecsys anti-Müllerian Hormone (AMH) fertility test, Roche) following the manufacturer’s instructions. The minimum detectable concentration of AMH was 0.01 ng/mL. Reagents and calibrators for the AMH analysis were supplied by the manufacturer. The standards ranged 0.01–23 ng/mL, and the test sensitivity was 0.14 ng/mL.

### Enumeration of ovarian follicles and morphological analysis

Mice were killed, and ovaries were harvested. All samples were washed thrice with phosphate-buffered saline (PBS), fixed with 4% paraformaldehyde (Sigma-Aldrich) for 30 min and stained with hematoxylin and eosin (HE) (Sigma-Aldrich) for histopathological examination under light microscopy. The number of healthy follicles at various stages of development was determined and follicles were classified as preantal, antral and atretic follicles as described previously (Reynier *et al.* 2001). The excised oocytes were enumerated by counting five random ovarian slides from five mice in each group. The average number of follicles on each slide was determined for statistical analysis.

### Ovulation induction and oocyte collection

Oocytes were prepared as described previously (Igarashi *et al.* 1997). After 8 weeks of rhGH administration, mice were superovulated with pregnant mare serum gonadotropin (PMSG, Sigma-Aldrich). After 48-h human chorionic gonadotropin (hCG) (Sigma-Aldrich) was injected intraperitoneally to recover mature (MII-stage) oocytes. For young mice (12 weeks old), 10 IU of both gonadotropins was administered, while aged mice (40 weeks old) received 15 IU of each. Oviducts were harvested from culled mice 14–16 h post hCG administration. Eggs were released into warmed M2 medium (Sigma-Aldrich) with 0.1% BSA (Sigma-Aldrich) by tearing oviducts with a 27-gauge needle. Cumulus cells were harvested via repeated aspiration with an attenuated glass transfer pipette and addition of 300 mg/mL hyaluronidase (Sigma-Aldrich). Cumulus-free oocytes were washed thrice with three drops of M2 without hyaluronidase and incubated at 37°C in 5% CO_2_ in air. Oocytes released from young and aged mice were randomly selected for subsequent analyses.

### Estimation of oocyte ATP levels

Every single MII-stage oocyte was placed in a sterile tube with 50 μL of PBS as a single sample and stored at −80°C until analysis. The average ATP content in each oocyte was quantified by measuring luminescence using a luminometer (SynergyHTX Multi-Mode Microplate Reader, BioTek) following the manufacturer’s instructions and a commercial assay kit (Beyotime Institute of Biotechnology). A standard curve including six ATP concentrations, ranging 0.32–200 nM, in the sample buffer was plotted for each series of the analyses. Samples were rapidly prepared to prevent enzymatic ATP hydrolysis. The contents of the sample tubes were transferred to a 96-well plate and the ATP concentration was determined using the formula derived from the linear regression of the standard curve.

### Immunofluorescence and mitochondrial staining

To specifically assess active mitochondrial distribution, ovulated MII-stage oocytes were incubated with prewarmed (37°C) 200 nM Mito Tracker Red FM (Invitrogen) for 20 min at 37°C, with 5% CO_2_ according to the manufacturer’s instructions. Oocytes were then washed thrice with M2 medium and fixed in 2% paraformaldehyde for 15 min. Subsequently, oocytes were incubated in Hoechst 33342 DPBS solution (2 μg/mL) for 10 min to stain DNA. Fixation and Hoechst 33342 staining procedures were performed at room temperature. For mitochondrial evaluation, samples were observed at 400× magnification using an Olympus FluoView1000 Laser Scanning Confocal Microscope (Olympus). MitoTracker Red FM fluorescence was measured using a 488-nm beam from a krypton-argon laser and a 594-nm band-pass filter. Images thus obtained were processed using Image J software (http://imagej.nih.gov/ij/, National Institutes of Health, Bethesda, MD, USA).

### Quantification of mitochondrial membrane potential via JC-1 staining

Mitochondrial membrane potential was quantified as described by Wilding *et al.* (2001). The mitochondrial probe, 5,5′,6,6′-tetrachloro-1,1′,3,3′-tetraethyl    benzimidazolyl-carbocyanineiodide (JC-1) is a dual-emission, potential-sensitive indicator that accumulates preferentially within mitochondria in the ooplasm (Reers *et al.* 1995). Oocytes were incubated in M2 medium containing JC-1 (10 μg/mL, Biovision, USA) at 37°C for 15 min. Thereafter, the distribution of JC-1 monomers (green fluorescence) and J-aggregate fluorescence (red fluorescence) was immediately observed using a confocal laser microscope. When in contact with membranes with high potential, JC-1 forms aggregates that emit fluorescence at 590 nm in response to 488 nm excitation. The captured images were processed using ImageJ software. Mitochondrial membrane potential was assessed by measuring the red:green fluorescence ratio. The area of measurement was adapted to the size of each oocyte, and the average intensity of every oocyte was considered for statistical analysis.

### Quantification of mtDNA copy number

Oocytes were transferred individually to a 0.2 mL tube containing 20 μL ddH_2_O. The contents were then freeze-thawed twice to lyse oocytes and release the DNA, which was stored at −80°C until use. qPCR primers for mouse mtDNA sequences (forward primer: CGAAAGGACAAGAGAAATAGAG; reverse primer: GAACAAGGTTTTAAGTCTTACGCA) were designed using Primer Premier 5.0 analysis software. The LightCycler 96 real time fluorescent quantitative PCR instrument (Roche) was used to determine the mtDNA copy number using SYBR Green Quantitative PCR kit (Takara) following the manufacturer’s protocol. PCR conditions were as follows: 95°C for 5 min and 40 cycles at 95°C for 30 s, 56°C for 25 s and 72°C for 20 s. All reactions were run twice, in triplicate. The SYBR green fluorescence was read at the end of each extension step. A melting curve was analyzed to determine the specificity of the PCR product. A standard curve was plotted with seven 10-fold serial dilutions of the external standard. The mtDNA copy number was determined from the standard curve.

### Statistical analysis

All experiments were replicated at least thrice independently. Analyses were performed using a Student’s *t*-test or one-way ANOVA using the SPSS, version 22.0 software (IBM). Mitochondrial distribution percentages were compared using the Chi-square test. mtDNA copy number and ATP content per oocyte data were untransformed. Data are presented as the means ± standard deviation (mean ± s.d.) values. Results were considered statistically significant if *P* < 0.05.

## Results

### Medium- and high-dose rhGH injection increased antral follicles of aged mice upon ovarian stimulation

**Figure 1:**
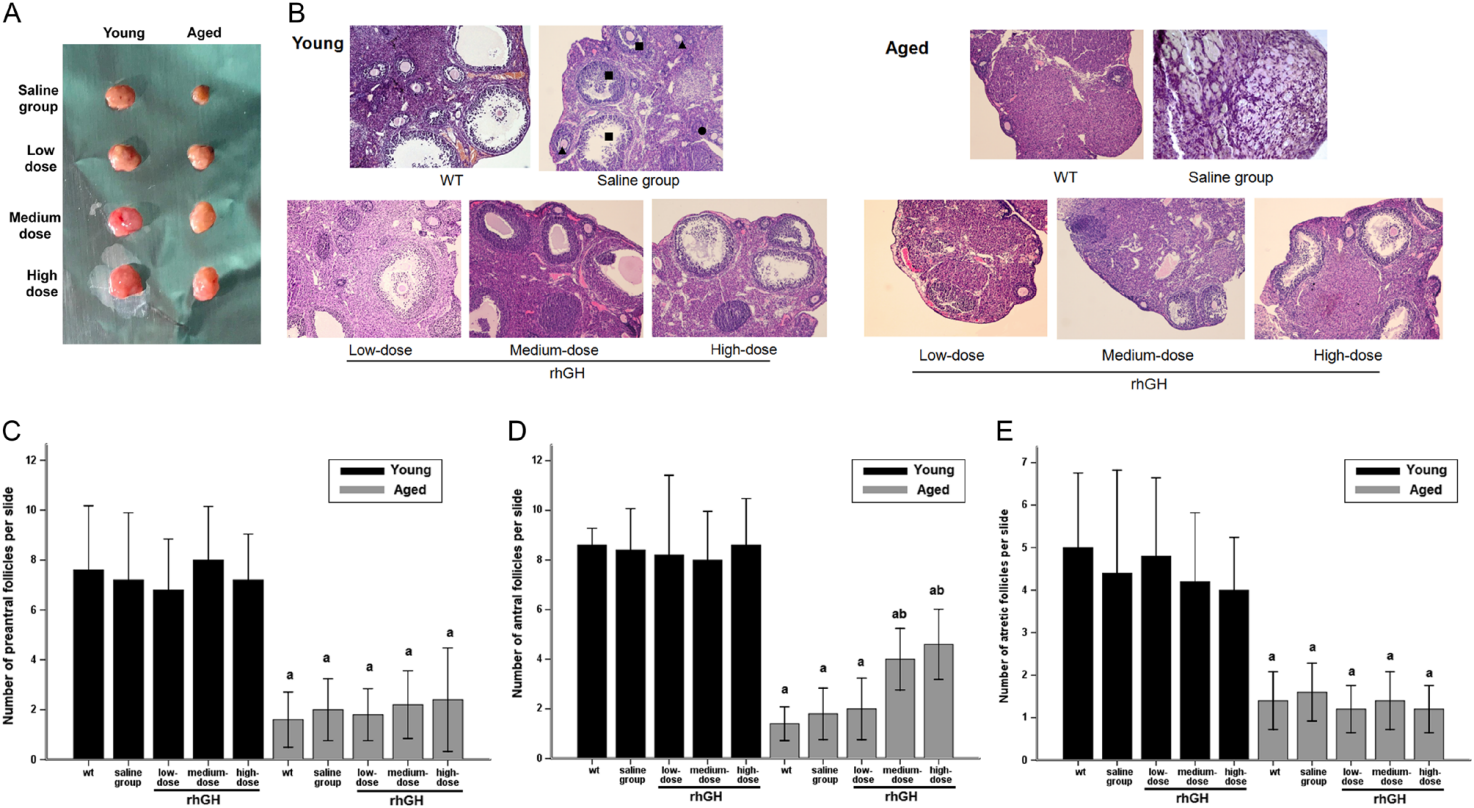
Impact of rhGH treatment on ovarian reserve and follicle counts in ovaries stained by hematoxylin and eosin. (A) Images of stimulated ovaries from each group are shown. Ovarian volume was significantly larger treated with rhGH for a period of 8 weeks compared with the saline group in aged mice. The difference was not obviously among the young mice groups. (B) Different types of ovarian follicles were observed: preantral follicle (solid, black triangle), antral follicle (solid, black square), atretic follicle (solid black circle). Magnification ×100. Scale bar: 200 µm. Ovarian reserve was not significantly improved after treated with rhGH either in young and aged mice groups because the number of resting preantral follicles was not higher. However, significantly higher number of growing antral follicles treated with medium/high-dose rhGH was observed in aged mice but not in young mice groups. Average number of follicles at various stages of development per slide was shown above: (C) preantral follicle, (D) antral follicle and (E) atretic follicle. ^a,b^Significantly different from control groups (^a^compared with wt/young group; ^b^compared with wt/aged group), respectively at *P* < 0.05 using one-way ANOVA analysis.

Ovary volume was measured for each group. Ovarian size did not differ significantly among young mice after 8 weeks of rhGH injection. However, ovaries of mice in the saline-treated group were significantly smaller than those of aged mice treated with different doses of rhGH (*P* < 0.05, Fig. 1A). HE staining revealed that the ovaries of young mice of all treatment and control groups contained numerous follicles at all developmental stages. However, the aged ovaries of WT/saline-treated mice and of the low-dose rhGH injection group primarily comprised interstitial cells in a fibrous matrix, with fewer follicles at each stage. Conversely, the ovaries of aged mice treated with medium- and high-dose rhGH for 8 weeks contained a significantly increased number of antral follicles (Fig. 1B). Following HE staining, the average number of normal (preantral and antral follicles) and atretic ovarian follicles in each group was determined on five randomly selected slides. At every developmental stage, the mean number of ovarian follicles of young mice was more than three-fold those in the ovaries of aged mice (*P* < 0.05). The number of resting preantral follicles did not increase in any group after rhGH treatment. Furthermore, the number of normal follicles and atretic follicles in the rhGH model and the WT/saline-treatment groups did not differ significantly in young mice (*P* > 0.05). Conversely, in aged mice, antral follicles were significantly more numerous in the medium- and high-dose groups than in the WT group (4.00 ± 1.00, 4.60 ± 1.14, and 1.40 ± 0.55 antral follicles in medium-dose, high-dose and WT aged mice, respectively; *P* < 0.05, Fig. 1D). The medium- and high-dose groups had slightly but not significantly more preantral follicles than the WT group, although this difference was not significant (2.20 ± 1.09, 2.40 ± 1.67, and 1.60 ± 0.89 preantral follicles in medium-dose, high-dose, and WT aged mice, respectively; *P* > 0.05, Fig. 1C). Additionally, the medium- and high-dose groups had slightly but not significantly fewer atretic follicles than the WT group, but this difference was not significant (1.40 ± 0.55, 1.20 ± 0.44, and 1.40 ± 0.54 atretic follicles in medium-dose, high-dose and WT aged mice, respectively; *P* > 0.05, Fig. 1E) (Supplementary Table 1, see section on supplementary data given at the end of this article).

### Serum AMH remained unchanged after rhGH injection for 8 weeks

**Figure 2:**
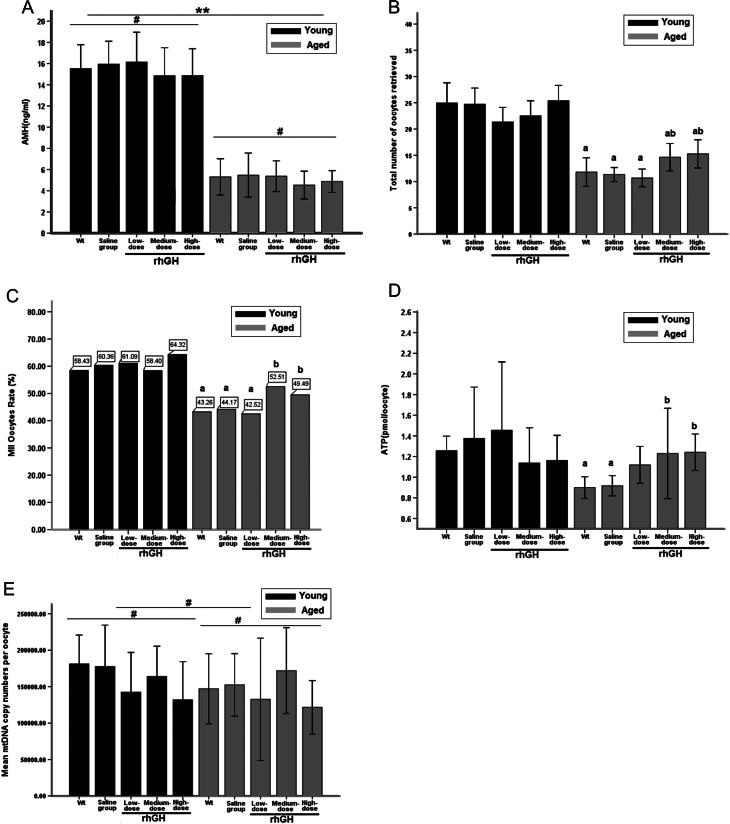
(A) Serum AMH levels following treatment with rhGH or saline (^#^ *P* > 0.05, vs Wt group of the same age group respectively; **P < 0.01, vs Wt group/young). (B) The average total number of ovulated oocytes collected after hormonal stimulation (^a^ *P* < 0.05, vs Wt group/young; ^b^ *P* < 0.05, vs Wt group/aged). (C) Formation rates of MII-stage oocytes (^a^ *P* < 0.05, vs Wt group/young; ^b^ *P* < 0.05, vs Wt group/aged). (D) ATP concentration of each single oocyte (^a^ *P* < 0.05, vs Wt group/young; ^b^ *P* < 0.05, vs Wt group/aged). (E) Mean mtDNA copy numbers per oocyte (^#^ *P* > 0.05, vs Wt group in both young and aged mice), most of the samples have approximately 170,000 copies. Descriptive statistics: minimum = 28,750; maximum = 537,664.

The average serum AMH levels in young mice (15.46 ± 3.39 ng/mL) was nearly 3-fold that in aged mice (5.11 ± 2.12 ng/mL) (Fig. 2A). Statistically significant difference between young and aged groups was expressed as *P* < 0.05 (Supplementary Table 2). However, after low-, medium- and high-dose rhGH treatment of aged mice for 8 weeks, serum AMH levels did not significantly differ from those observed in the WT/saline model groups (*P* > 0.05) and remained unchanged in young mice.

### Medium- and high-dose rhGH increased retrieved oocyte numbers and promoted oocyte maturation in aged mice

The average number of oocytes retrieved from aged mice (12.65 ± 5.78) was nearly half that from young mice (23.69 ± 7.62, *P* < 0.05). Similar results were observed for MII-stage oocytes (6.08 ± 4.19 and 14.48 ± 8.37 in aged and young mice, respectively, *P* < 0.05). Medium- and high-dose rhGH treatment significantly increased the total number of retrieved oocytes, MII-stage oocytes and MII-stage oocyte rate in aged mice rather than in the WT/saline groups (Fig. 2B and C, *P* < 0.05, Supplementary Table 2). No significant difference was observed between the low-dose group and the WT/saline control groups (*P* > 0.05). Formation rates of MII-stage oocytes from aged mice treated with low-dose rhGH (42.52%) and WT (43.26%) and saline (44.17%) control groups were significantly lower than those in young mice (58.40–64.32%, *P* < 0.05). Furthermore, MII-stage oocyte rates in aged mice after treatment with medium- (52.51%) and high-dose (49.49%) rhGH were slightly but not significantly lower than the average rate of all the young groups (59.11%) (*P* > 0.05). Medium- and high-dose rhGH injections were suggested to improve oocyte maturation in aged mice to a level similar to that observed in young mice. Although the MII-stage oocytes rate in the young mice high-dose group was the highest observed (64.32%), no significant difference was observed between any of the young mice rhGH treatment and control groups (*P* > 0.05).

### Medium- and high-dose rhGH treatment increased ATP levels in aged mice

The ATP standard curve was linear and very sensitive. ATP levels from single oocyte were interpolated from the curve. The ATP levels in single oocytes from aged WT and saline groups (0.89 ± 0.10 and 0.92 ± 0.09 pmol/oocyte, respectively) were significantly lower than those of young mice (average level: 1.28 ± 0.44 pmol/oocyte, *P* < 0.05) (Fig. 2D). However, medium- and high-dose rhGH treatment in aged mice significantly increased ATP levels (1.23 ± 0.47 and 1.24 ± 0.17 pmol/oocyte following medium- and high-dose treatments, respectively) (*P* < 0.05). ATP levels did not differ significantly upon low-dose treatment between aged mice and the control groups. In young mice, oocyte ATP levels after medium- (1.14 ± 0.37 pmol/oocyte) and high-dose (1.16 ± 0.20 pmol/oocyte) rhGH treatment did not differ significantly from the WT (1.26 ± 0.13 pmol/oocyte) and saline groups (1.38 ± 0.59 pmol/oocyte, *P* > 0.05). Furthermore, ATP levels of oocytes retrieved from aged mice after medium- (1.23 ± 0.47 pmol/oocyte) and high-dose (1.24 ± 0.17 pmol/oocyte) rhGH treatment were slightly but not significantly lower than that of the young mice WT and saline control groups (1.26 ± 0.13 and 1.37 ± 0.59 pmol/oocyte), no significant difference was observed (*P* > 0.05). These results suggest that medium- and high-dose rhGH treatment enabled sufficient ATP output for oocyte maturation in aged mice and increased ATP generation to a level similar to that observed in the young controls.

### Medium- and high-dose rhGH treatment increased the frequencies of homogeneous mitochondrial distribution in aged mice

**Figure 3:**
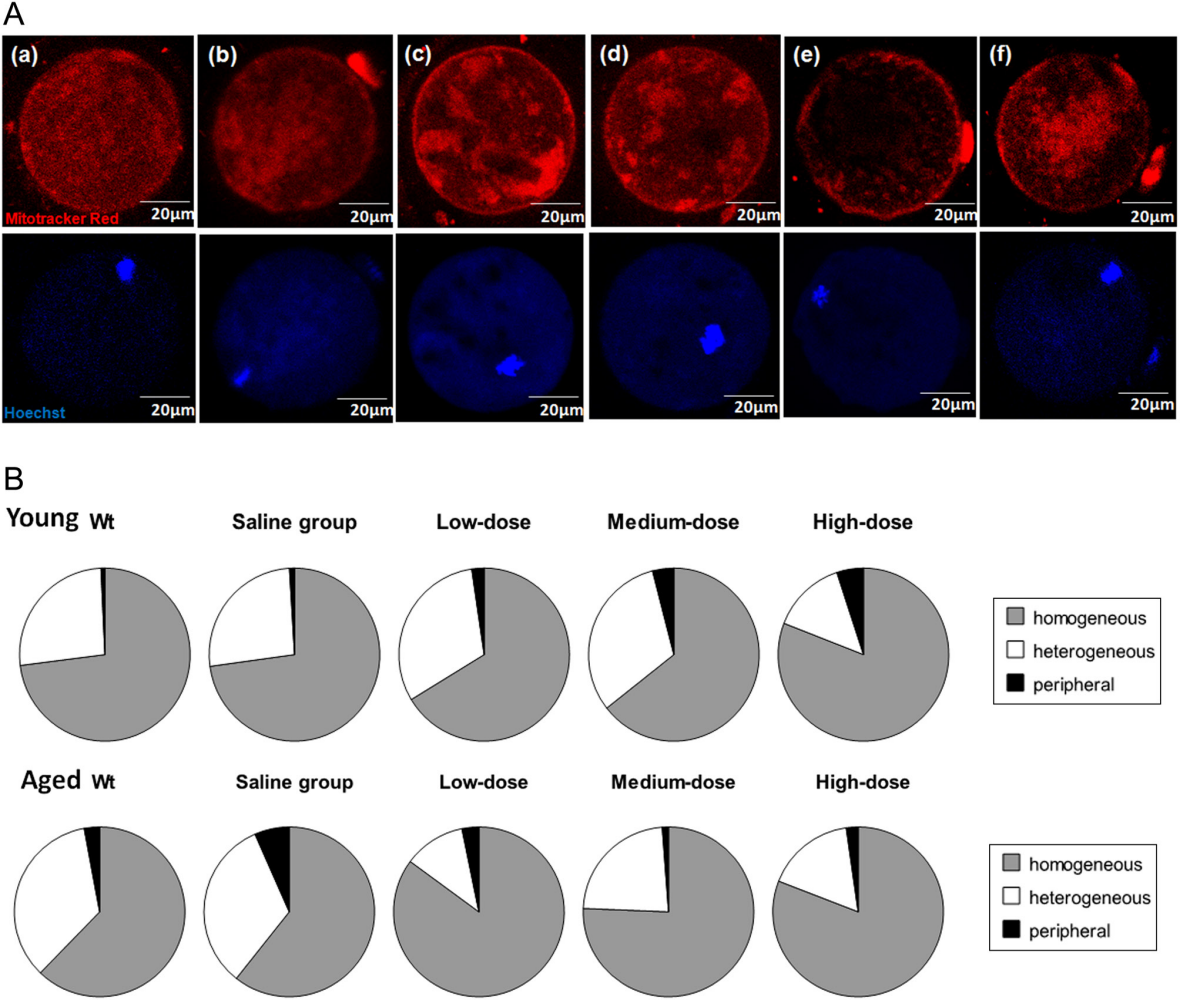
(A) Representative photographs of mouse oocytes were obtained from confocal microscopy, (a, b, c, d, e and f) illustrate the different types of mitochondrial distribution: (a and b) homogeneous mitochondrial distribution throughout the entire oocyte; (c and d) heterogeneous-mitochondria distributed heterogeneously with aggregation, large granules distributed throughout the ooplasm; (e and f) peripheral mitochondrial in the periphery or central ooplasm of the oocyte. Original magnification 400×, white bars represents 20 µm. Staining by MitoTracker@Red FM. (B) Percentages of three mitochondria distribution types of the oocytes retrieved from the young and aged mice in different interventional groups.

The mitochondrial distribution pattern was observed via MitoTracker@Red FM staining. According to Brevini, confocal microscopy images show three different patterns of mitochondrial distribution (Brevini *et al.* 2005). Oocytes presented one of the following patterns: (i) homogeneous mitochondrial, distributed throughout the entire oocyte; (ii) heterogeneous-mitochondrial, distributed heterogeneously with aggregation, large granules distributed throughout the ooplasm; (iii) peripheral mitochondrial, in the periphery or central ooplasm of the oocyte (Fig. 3A). The most common pattern observed in 51.3–60.0% of oocytes from young mice and in 62.1–66.7% of oocytes from rhGH treatment groups in aged mice was a homogeneous granular pattern with large mitochondrial aggregates distributed throughout the cytoplasm (Fig. 3B). This pattern was observed in significantly fewer oocytes from aged mice of the WT (41.7%) and saline groups (35.1%) (*P* < 0.05, compared with both the WT/young group and the rhGH-treated aged groups, Supplementary Table 3). Furthermore, the percentages of heterogeneous and peripheral mitochondrial distribution in oocytes retrieved from aged mice treated with low-dose (25.0 and 12.5%, respectively), medium-dose (29.2 and 4.1%, respectively) and high-dose (31.0 and 6.9%, respectively) rhGH for 8 weeks were significantly lower than those of the WT (38.9 and 19.4%, respectively) and saline-treated groups (31.6 and 33.3%, respectively) (*P* < 0.05). The frequencies of homogeneous mitochondrial distribution in aged mice treated with rhGH were slightly but not significantly higher than those observed in the WT/young group (*P* > 0.05).

### Medium- and high-dose rhGH injection improved mitochondrial membrane potential in aged and young mice

**Figure 4:**
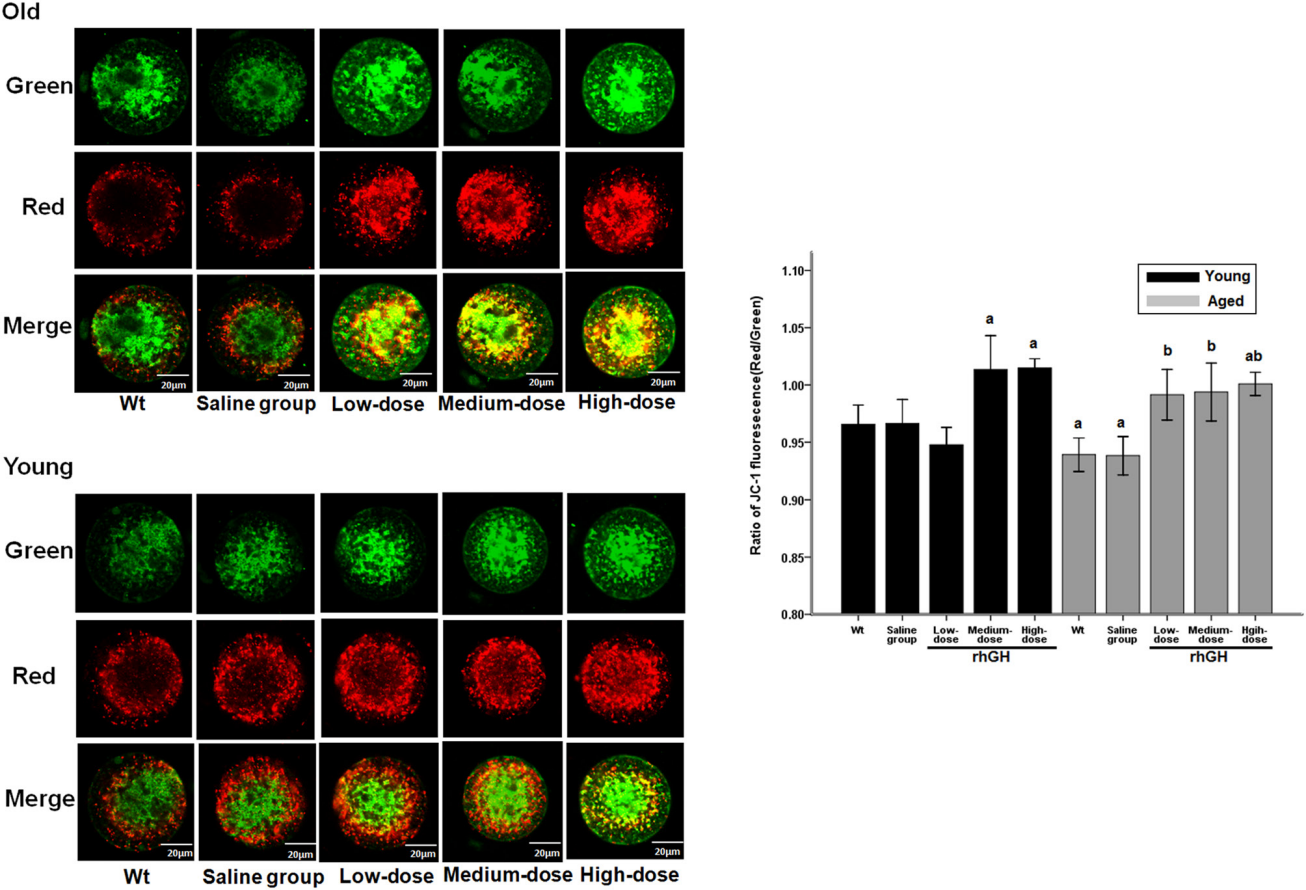
Representative pictures show oocytes of young mouse and aged mouse with/without rGH treatment stained with JC-1. Green and red fluorescence indicates JC-1 monomer and JC-1 aggregate fluorescence, respectively. Bar, 20 μm. Ratios of red/green JC-1 fluorescence in young, aging mouse and low, medium, high dose of rGH-treated oocytes were represented. The results are shown as the mean ± s.d. All experiments were performed in at least three independent runs. Bars with different letters represent significant differences. ^a^ *P* < 0.05, compared with wt/young group. ^b^ *P* < 0.05, compared with wt/old group.

Ratios of red/green JC-1 fluorescence intensity, reflecting mitochondrial membrane potentials (ΔΨm), were determined for every oocyte of each group. Green fluorescence represents the monomeric form of JC-1, indicating dissipation of ΔΨm. Red fluorescence indicates mitochondrial aggregate forms of JC-1, indicating intact mitochondrial membrane potential. Compared with red fluorescence staining peripherally distributed in MII-stage oocytes, green fluorescence staining was relatively centrally distributed. All rhGH treatment groups (low, medium and high dose) significantly increased the total ratios of red/green JC-1 fluorescence intensity (0.991 ± 0.018, 0.994 ± 0.016 and 1.001 ± 0.008, respectively) of MII-stage oocytes from aged mice compared with WT group mice (0.938 ± 0.016, *P* < 0.05). Similarly, after treatment with medium- and high-dose rhGH, the ratios of red/green JC-1 fluorescence intensity were significantly increased in the oocytes of young mice (1.014 ± 0.035 and 1.015 ± 0.008) rather than in those of WT mice (0.966 ± 0.023, *P* < 0.05). No significant difference was observed between low-dose treatment and WT groups (*P* > 0.05). The present results indicate that the pool of respiring mitochondria was low in aged oocytes but increased upon rhGH treatment to levels similar to, or higher (high-dose group, *P* < 0.05) than, those of the young controls (Fig. 4). No significant changes in mitochondrial membrane potential (ΔΨm) were observed in young or aged oocytes exposed to saline.

### mtDNA copy number did not differ significantly in all groups

The mtDNA copy numbers varied among oocytes. After treatment with low-, medium- and high-doses of rhGH for 8 weeks, the average mtDNA copy number per oocyte did not change significantly compared with those observed in the WT/saline control groups (Fig. 2E). This held true for both young and aged mice (young mice median: 147,113; IQR: 98,228–241,449; aged mice median: 124,672; IQR: 80,234–204,589, *P* > 0.05). The mtDNA copy number of single oocytes retrieved from aged mice treated with medium-dose rhGH (mean: 197,073 copies) was slightly but not significantly higher than those of single oocytes from the WT/saline control groups (*P* > 0.05; WT mean: 180,197; saline mean: 175,789). Comparison the average mtDNA copy number per oocyte from young WT/saline control mice and the medium-dose rhGH treatment group revealed that copy numbers of mtDNA were similar (control mean: 200,213; medium-dose mean: 207,567, *P* > 0.05) (Supplementary Table 4).

## Discussion

Reproductive efficiency decreases with increasing maternal age. Clinical results support a decline in response to ovarian stimulation during IVF, poor oocyte and embryo developmental competence increased incidence of spontaneous pregnancy loss and fetal aneuploidy in older infertility patients. The primary mechanism believed to underlie the reduction in oocyte quality is the increased aneuploidy rate. Mitochondrial functionality is a hallmark of the quality and developmental potential of oocytes (Dumollard *et al.* 2004, Van Blerkom 2004, Dumollard *et al.* 2009, Van Blerkom 2009). Aberrant mitochondrial activity reduces ATP production, is involved in meiotic chromosomal nondisjunction and may limit cell division and embryonic development (Hassold & Chiu 1985b, Antonarakis *et al.* 1992, Munne *et al.* 1995, Dailey *et al.* 1996, Yu *et al.* 2010, Bentov *et al.* 2011, Simsek-Duran *et al.* 2013, Dalton *et al.* 2014). As mammalian mitochondria are maternally transmitted, healthy and high-quality oocytes indicate higher fertilization ability and embryonic developmental potential (Santos *et al.* 2006). However, higher mtDNA copy numbers indicate a reduced embryonic developmental potential. Supplementation with mitochondrial nutrients can successfully alleviate conditions associated with diminished mitochondrial energy production, and mitochondrial nutrients appear safe for both the mother and the fetus. Increasing the energy available for chromosomal disjunction may improve oocyte and embryo quality and a better pregnancy outcome among older women.

Oocyte growth depends on interactions with granulosa cells (GCs) that provide energy to oocytes (Orisaka *et al.* 2009), and high granulosa cellular glycolytic activity is important for folliculogenesis (Munakata *et al.* 2016). During oocyte maturation, the mitochondrial membrane is seemingly regulated by cumulus GCs surrounding the oocyte (Van Blerkom 2009), reflecting the functional diversity of oocyte mitochondria. GH is a pleiotropic hormone that affects various physiological functions, from carbohydrate and lipid metabolism to the immune response (Chesnokova *et al.* 2013, Visser *et al.* 2013, Cecchi *et al.* 2014, Kopchick *et al.* 2014, Rojanathammanee *et al.* 2014). GH receptor expression within GCs of human Graafian follicles declines with age (Regan *et al.* 2012). Expression of GH receptor mRNA in the ovaries of hypophysectomized rats was significantly increased after treatment with GH, suggesting that GH upregulates its own receptor expression. Similarly, a recent study reported that short-term GH action promotes mitochondrial oxidative capacity within skeletal muscle cells (Short *et al.* 2008). GH reported regulates the effects of FSH on GCs and promotes granulosa cell proliferation in preantral follicles of mice (Kobayashi *et al.* 2000). rhGH treatment improves oocyte quality by upregulation of its own receptors and by enhancing mitochondrial activity (Weall *et al.* 2015).

rhGH has been used as an adjunct for ovarian stimulation for women with a previous poor ovarian response (POR) to stimulation in an IVF cycle for 25 years. Recent studies support rhGH supplementation for clinically managing POR patients. Most studies have reported that rhGH treatment increases ovarian sensitivity to exogenous gonadotropin (Gn) stimulation, increases MII-stage recovery (Bassiouny *et al.* 2016), the fertilization rate (Kucuk *et al.* 2008), the number of high-quality embryos and the probability of pregnancy (Ob’Edkova *et al.* 2017). One of the most extensive analyses of the role of rhGH in IVF over a period of 7.5 years reported that rhGH clinically increased pregnancy rates by 3.42-fold (95% CI 1.82–6.44) and live birth rates by 6.16-fold (95% CI 2.83–13.39) in poor responders after adjusting maternal age, antral follicle count and transferred embryo quality (Keane *et al.* 2017). Nonetheless, the applicability of rhGH co-treatment in IVF cycles for POR patients remains controversial (de Ziegler *et al.* 2011, Hart *et al.* 2017). A randomized controlled trial (RCT) reported that rhGH administration significantly increased the number of collected oocytes, metaphase II oocytes and transferred embryos, but not the clinical pregnancy rate and live birth rate (Bassiouny *et al.* 2016). However, most systematic reviews and meta-analyses suggested that rhGH administration significantly increased both clinical pregnancy rate and live birth rates (Duffy *et al.* 2010). Moreover, the efficacy of rhGH co-treatment in IVF cycles in normo-responder patients with at least three or more failed embryo transfers for no discernible reasons was investigated. Co-stimulation with 8 IU rhGH yielded better results in terms of the number of harvested oocytes and embryos. Similarly, the present study shows that medium- and high-dose rhGH co-treatment increased the number of total harvested oocytes and MII-stage oocytes. The oocyte maturation rate of aged mice treated with medium- and high-dose rhGH was similar to that of young mice.

The mechanism underlying rhGH-mediated improvement of pregnancy outcomes was presumed to be embryo mediated or endometrium mediated. Cui *et al.* reported that rhGH may improve endometrium thickness on day 3, implantation rates and clinical pregnancy rates of patients with thin endometria who underwent frozen embryo transfer via promotion of proliferation and vascularization and upregulation of receptivity-related gene expression (Cui *et al.* 2018). Another randomized clinical trial enrolled patients with a history of repeated implantation failure (RIF) upon oocytes transplantation to determine whether rhGH administration can improve the chance of pregnancy and birth and reported that RIF patients administered GH had significantly thicker endometrial and higher chances of pregnancy and live birth rates. Improvement in cytoplasmic competence has been proposed as an explanation.

The exact mechanism by which GH action regulates oocyte mitochondrial function remains unknown, as do considerations related to GH dosage. In clinical studies, protocols for GH administration are heterogeneous. Interventional doses of rhGH vary from 2 IU to 12 IU daily or from 4 IU to 24 IU on alternate days before or during the period of COH in different IVF facilities, with differing outcomes (Li *et al.* 2017). In most of the studies, rhGH is usually administered via daily subcutaneous injection starting from day 1 of ovarian stimulation until the day of hCG triggering. Another strategy was 4- to 6-week pretreatment before day 1 of ovarian stimulation. rhGH pretreatment improved embryo quality and significantly decreased miscarriages thus increasing the live birth rate. Longer term use of low-dose rhGH (2 IU/day) administration for 6 weeks may benefit the utilization of oocytes and to finally increase live birth rates of POR (Cai *et al.* 2018). Herein, we administered rhGH at three different interventional rhGH injection doses (0.4, 0.8 and 1.6 mg/kg/day) to determine the most effective dosage for aged female mice. For clinical use for older infertility patients, the doses used here can be converted to 5, 10 and 20 IU/day for low-, medium- and high-dose regimens, respectively. The present results show that rhGH supplementation at 0.8 and 1.6 mg/kg/day for 8 weeks significantly increased the total oocyte number and the number of MII-stage oocytes retrieved via superovulation. Medium-dose rhGH can improve oocyte mitochondrial function but does not increase mitochondrial quantity. This result supports the development of strategies to increase oocyte quality using mitochondrial nutrients (Bentov *et al.* 2010, 2011, 2014).

AMH is directly produced by ovarian GCs of preantral and early antral follicles up to 6 mm in diameter (Broekmans *et al.* 2008). Serum AMH levels correlate with the follicular pool and appear to be the earliest endocrine marker of ovarian aging (van Rooij *et al.* 2002, Weenen *et al.* 2004, Lie *et al.* 2012). Serum AMH can be measured at any point during the menstrual cycle as its levels do not change significantly. The present results are concurrent with previous reports, showing a significant negative correlation between serum AMH and age (La Marca *et al.* 2009). In the present study, the average serum AMH levels measured in mice from the young groups were three-fold those in aged mice. A similar tendency in the mean number of follicles was detected at all observed stages. Furthermore, we did not observe a significant difference in preantral follicle numbers between rhGH treatment groups and control groups. These results verified that AMH level is a good predictor of oocyte quantity, and that rhGH treatment does not improve ovarian reserves. Interestingly, treatment with medium- and high-dose rhGH improved the ovarian follicular populations of antral follicles in aged mice when compared with the control group. The potential underlying mechanism probably involves the action of rhGH on follicle recruitment proceeding to terminal growth and its effect on follicle survival and growth (Bachelot *et al.* 2002). In addition, rhGH appears to directly or indirectly protect antral follicles from undergoing atresia and promotes follicular maturation to the preovulatory pool (Lucy 2000).

The ATP consumption rate is increased in the mature oocyte which is essential for its ability to undergo normal fertilization. Many mitochondrial functions, including ATP generation, protein import and lipid biogenesis, depend on the maintenance of membrane potential (ΔΨm). ATP synthesis in mouse oocytes decreases with age (Selesniemi *et al.* 2011, Simsek-Duran *et al.* 2013) and mitochondrial ΔΨm was highly correlated with the mitochondrial capacity for ATP synthesis (Dumollard *et al.* 2007). In the present study, oocyte ATP levels of oocyte were significantly lower in aged mice than in young mice. Oocyte mitochondria energy production decreases with age during IVF cycles and is associated with increased aneuploidy rates. rhGH administration may improve mitochondrial function and oocyte developmental potential (Weall *et al.* 2015). We assessed the effects of three different rhGH concentrations on oocytes retrieved from aged and young mice. In aged mice, higher ATP concentrations were observed with medium- and high-dose rhGH Mitochondrial membrane potential (ΔΨm) is closely associated with ATP synthesis and embryonic development after fertilization (Wilding *et al.* 2001, Van Blerkom *et al.* 2006). Disrupted mitochondrial distribution is considered to cause oocyte aging. Therefore, we investigated whether rhGH treatment improves oocyte mitochondrial performance. The present results suggest that both mitochondrial membrane potential (ΔΨm) and percentages of homogeneous granular pattern of mitochondrial in oocytes from rhGH treatment groups were significantly improved in aged mice. Immunofluorescence staining experiments confirmed higher mitochondrial immunoreactivity in rhGH treatment groups than in the control group in aged mice, indicating that rhGH enhances mitochondrial function. These results reasonably suggest that medium- and high-dose rhGH promotes oocyte maturation and improves oocyte quality in aged mice.

Other than the nucleus, mitochondria are the only organelles containing DNA. The oocyte has the largest number of mitochondria and mtDNA copies of any cell type including muscle and neurons cells, which have high energy demands. Oocyte mitochondria contain only one or two mtDNA genomes (Jansen 2000, Reynier *et al.* 2001, May-Panloup *et al.* 2007). Therefore, mitochondrial quantity in a single human egg can be estimated through the mtDNA copy number ranging 138,000–640,000. However, the relationship between oocyte mitochondrial function and mtDNA copy number in oocytes remains unclear. Considerable inter-oocyte variability in mtDNA content has been reported. mtDNA replication occurs constantly as the oocyte matures and ceases after maturation. MII-stage oocytes can contain 50,000–1,500,000 copies of mtDNA (Monnot *et al.* 2013). In the present study, qPCR analysis of individual mature oocytes revealed an average mtDNA copy number of 180,000 molecules, which is somewhat higher than the 119,000 copies/oocyte previously reported by Piko and Taylor (Piko & Taylor 1987). A range of concentrations, from 80,000 to 290,000 copies/oocyte, was detected among the samples examined by Piko and Taylor (Piko & Taylor 1987). However, a higher degree of variability was noted among the present samples (28,000–540,000 copies/oocyte).

Fragouli *et al.* (2015) reported that the number of mitochondrial genomes appeared decreased with advanced maternal age, whereas Diez-Juan *et al.* (2015) did not report a correlation with maternal age. Oocytes of women with ovarian insufficiency reportedly have lower mtDNA copy numbers than those of women with a normal ovarian profile (May-Panloup *et al.* 2005, 2016). However, other studies reported no significant difference in mtDNA copy number between patients with diminished ovarian responses and those with normal ovarian responses. In the present study, we observed similar mtDNA copy numbers in young and aged mice. Together, these results suggest that it is mitochondrial function, and not mitochondrial number, is affected by maternal age. mtDNA copy number is proposed to be a consequence of the oocyte’s energy status, not a cause, and as such is a poor biomarker for mitochondrial content (Larsen *et al.* 2012).

It has been speculated that alterations in mtDNA copy number do not affect oocyte maturation but correlate with the ability of oocytes to be fertilized. Unfertilized oocytes have lower mtDNA copy numbers (Reynier *et al.* 2001, Santos *et al.* 2006). However, the total number of mtDNA copies in the developing embryo does not change from fertilization until the blastocyst stage (Thundathil *et al.* 2005, Houghton 2006). Clinical studies have reported that high mtDNA copy number in euploid embryos indicates lower embryo viability in terms of implantation potential (Diez-Juan *et al.* 2015). Interestingly, reductions in mtDNA copy number did not compromise ovulation and fertilization; however, post-implantation development was impeded when embryos had with fewer than 50,000 mtDNA copies (Wai *et al.* 2010), indicating that there is a threshold number of functional mitochondria required for normal development. Genetic manipulation of mtDNA copy number in mice revealed that the threshold oocyte mtDNA copy number required for embryonic development following implantation was 40,000–50,000. Too few mitochondria impede energy generation and possibly embryo quality, and too many mitochondria compromise cellular function (Wai *et al.* 2010). mtDNA copy number may be a consequence rather than the root cause of the oocyte’s energy status (Diez-Juan *et al.* 2015).

Interestingly, all groups pretreated with rhGH had significantly lower mtDNA copy numbers than the control groups in the young mice, although the difference was not significant. This result is concurrent with the concept that the mtDNA copy number and ATP content in the oocyte are not directly related (May-Panloup *et al.* 2007). Data from transmission electron microscopy and extrapolated predictions of actual mitochondrial numbers do not always correlate with mtDNA copy number estimates (Van Blerkom 2011). It is conceivable that the number of mtDNA copies per oocyte is not associated with mitochondrial number. Three explanations have been provided for this result. Firstly, energy production and mtDNA do not share a particularly healthy relationship. The mechanism underlying mitochondrial energy production involves the release of oxygen free radicals, which are short-lived but powerful oxidizing agents, and all material near the site of oxygen free radical release are targets for oxidation, including the proteins of the oxidative phosphorylation complex and the mtDNA. Moreover, a partial or no mechanism has been reported for mtDNA repair within the mitochondria. Secondly, improved mitochondrial function may reduce the mtDNA copy number. Increased mtDNA levels result from a compensatory mechanism in oocytes harboring functionally deficient mitochondrial organelles, which require more energy. Dysfunctional mitochondria are cleared by an autophagocytic process called mitophagy (Tolkovsky 2009), which depends on energy and on proper functioning of a minimal number of mitochondria. Lastly, mitochondrial inheritance is maternal, implying that although the egg contributes half of the genome, it contributes 100% of the mitochondrial content of the neonate. The ‘mitochondrial bottleneck’ hypothesis postulates that mitochondria with the most-fit mtDNA are selected for while possibly mutated mtDNA is eliminated to prevent mutations from being transmitted to the embryo (Reynier *et al.* 2001). rhGH pretreatment may decrease the mtDNA copy number threshold required for fertilization and embryonic development in oocytes. Whether rhGH is involved in the selection of mitochondria or reduces the mtDNA pool to a few copies to decrease mtDNA mutations requires further study.

In conclusion, the medium rhGH dose administration significantly enhances the MII-stage oocyte rate, ATP content and mitochondrial membrane potential in oocytes of aged mice. However, ovarian reserves and mtDNA copy number were not significantly increased in any group. In young mice, most of the evaluated parameters were not significantly altered except for mitochondrial membrane potential. The number of mtDNA copies is of substantial biological and clinical relevance. This requires further validation and our current understanding is inadequate. This study therefore demonstrates, for the first time, that medium-dose rhGH supplementation for 8 weeks before standard ovarian stimulation regimens improves oocyte quality in aged females, potentially by enhancing mitochondrial functionality. However, additional large-scale studies on rhGH dosing, length of pretreatment and safety of clinical outcomes are necessary before its clinical use.

## Supplementary Material

Supplementary Table 1

Supplementary Table 2

Supplementary Table 3

Supplementary Table 4

## Declaration of interest

The authors declare that there is no conflict of interest that could be perceived as prejudicing the impartiality of the research reported.

## Funding

This study was supported by the National Natural Science Foundation of China (81273977, 81402691, and 81528020) and the Natural Science Foundation of Tianjin (15JCQNJC12300, 12JCYBJC16200, and 15JCZDJC36000).
