# Supplementary material for: Evidence that growth hormone can improve mitochondrial function in oocytes from aged mice
Source: Reproduction. 2019 Jan 21;157(4):345–58. doi: 10.1530/REP-18-0529 (PMC6420407; doi:10.1530/REP-18-0529)
Supplement: Supplementary Table 1 [file supplementary_table_1.pdf]

Supplemental Table 1 Effect of rhGH on different classes of ovarian follicles in each group (average number per slide)

Treated groups	Preantral follicles	Antral follicles	Atretic follicles
**Young**			
Wt	7.60±2.07	8.60±0.55	5.00±1.41
Saline group	7.20±2.17	8.40±1.34	4.40±1.95
Low-dose/ rhGH	6.80±1.64	8.20±2.59	4.80±1.48
Medium-dose/rhGH	8.00±1.73	8.00±1.58	4.20±1.30
High-dose/rhGH	7.20±1.48	8.60±1.52	4.00±1.00
Total	7.36±1.73	8.36±1.52	4.48±1.39
**Aged**			
Wt	1.60±0.89 [a]	1.40±0.55 [a]	1.40±0.54 [a]
Saline group	2.00±1.00 [a]	1.80±0.84 [a]	1.60±0.55 [a]
Low-dose/ rhGH	1.80±0.84 [a]	2.00±1.00 [a]	1.20±0.45 [a]
Medium-dose/rhGH	2.20±1.09 [a]	4.00±1.00 [ab]	1.40±0.55 [a]
High-dose/rhGH	2.40±1.67 [a]	4.60±1.14 [ab]	1.20±0.44 [a]
Total	2.00±1.08 [a]	2.76±1.56 [a]	1.36±0.49 [a]

Note: Preantral follicles include primordial follicle, primary follicle and secondary follicle. Antral follicles inculde teritary follicle and mature follicle. Data expressed as Mean ±SD of 5 random ovarian slides from five females in each group. a, b significantly different from control groups(a: compared with wt/young group; b: compared with wt/aged group),respectively at *P*<0.05 using one-way ANOVA followed by Tukey-Kramer as a post-hoc test.
