# Supplementary material for: Evidence that growth hormone can improve mitochondrial function in oocytes from aged mice
Source: Reproduction. 2019 Jan 21;157(4):345–58. doi: 10.1530/REP-18-0529 (PMC6420407; doi:10.1530/REP-18-0529)
Supplement: Supplementary Table 2 [file supplementary_table_2.pdf]

Supplemental Table 2 Serum AMH level and collected MII oocytes in different groups (Mean ±SD)

Groups	Mice number	AMH(ng/ml)	Oocytes	MII oocytes	MII oocyte rate（%）
**Young**					
Wt	28	15.95±3.02	24.18±7.68	14.36±7.62	58.43
Saline group	29	15.52±3.15	25.00±8.58	15.46±8.65	60.36
Low-dose/ rhGH	25	16.14±3.95	21.36±6.71	13.76±8.77	61.09
Medium-dose/rhGH	27	14.84±3.69	22.52±7.26	13.07±5.53	58.40
High-dose/rhGH	23	14.86±3.55	25.39±6.78	16.91±8.82	64.32
Total	132	15.46±3.39	23.69±7.62	14.48±8.37	59.11
**Aged**					
Wt	26	5.47±2.92	11.36±5.78 [a]	5.13±4.22 [a]	43.26[a]
Saline group	26	5.11±2.12[a]	11.58±5.26 [a]	5.29±3.73 [a]	44.17[a]
Low-dose/ rhGH	29	5.37±2.02[a]	10.69±4.44 [a]	4.59±3.07 [a]	42.52[a]
Medium-dose/rhGH	24	4.53±1.83[a]	14.63±6.23 [abc]	7.83±5.27 [abc]	52.51[b]
High-dose/rhGH	25	4.87±1.44[a]	15.28±6.52 [abc]	7.76±4.16 [abc]	49.49[b]
Total	130	5.11±2.12 [d]	12.65±5.78 [d]	6.08±4.19 [d]	46.39 [d]

Note: Different superscript letters within columns represent significant differences with groups (*P*<0.05, [a]compared with wt/young group, [b]compared with wt/old group, [c]compared with aged Low-dose group, [d]compared with total outcomes of the young group).
