# Supplementary material for: Evidence that growth hormone can improve mitochondrial function in oocytes from aged mice
Source: Reproduction. 2019 Jan 21;157(4):345–58. doi: 10.1530/REP-18-0529 (PMC6420407; doi:10.1530/REP-18-0529)
Supplement: Supplementary Table 3 [file supplementary_table_3.pdf]

Supplemental Table 3　Distribution of mitochondria

Oocytes	Homogeneous%(n)	Heterogeneous%(n)	Peripheral%(n)
**Young**			
Wt	58.8(40/68)	35.3(24/68)	5.9(4/68)
Saline group	58.3(35/60)	35.0(21/60)	6.7(4/60)
Low-dose/ rhGH	53.3(48/90)	36.7(33/90)	10.0(9/90)
Medium-dose/rhGH	51.3(40/78)	35.9(28/78)	12.8(10/78)
High-dose/rhGH	60.0(48/80)	25.0(20/80)	15.0(12/80)
**Aged**			
Wt	41.7(30/72)^{a}	38.9(28/72)^{a}	19.4(14/72)^{a}
Saline group	35.1(20/57)^{a}	31.6(18/57)^{a}	33.3(9/57)^{a}
Low-dose/ rhGH	62.5(35/56)^{b}	25.0(14/56)^{b}	12.5(7/56)^{b}
Medium-dose/rhGH	66.7(32/48)^{b}	29.2(14/48)^{b}	4.1(2/48)^{b}
High-dose/rhGH	62.1(36/58)^{b}	31.0(18/58)^{b}	6.9(4/58)^{b}

Note: Different superscript letters (a,b) within columns represent significant differences with groups(P<0.05, ^{a}compared with wt/young group, ^{b}compared with wt/old group).
