# Supplementary material for: Evidence that growth hormone can improve mitochondrial function in oocytes from aged mice
Source: Reproduction. 2019 Jan 21;157(4):345–58. doi: 10.1530/REP-18-0529 (PMC6420407; doi:10.1530/REP-18-0529)
Supplement: Supplementary Table 4 [file supplementary_table_4.pdf]

Supplemental Table 4 The mtDNA characteristics in the experimental groups (Mean)

Groups	Number of analyzed oocyte	Mean mtDNA copy number ±SD	Range
**Young**			
Wt	130	200 213±139 367	29 985-537 664
Saline group	138	207 567±141 173	28 750-479 553
Low-dose/ rhGH	126	158 013±124 895	38 956-418 850
Medium-dose/rhGH	119	191 681±124 964	70 346-499 637
High-dose/rhGH	129	144 516±119 223	28 843-387 051
Total	642	181 330±127 268	28 750-537 664
**Aged**			
Wt	63	180 197±136 017	38 711±498 440
Saline group	60	175 789±115 665	54 866±438 847
Low-dose/ rhGH	73	156 583±137 261	50 223±399 456
Medium-dose/rhGH	75	197 073±140 483	59 866±488 563
High-dose/rhGH	67	144 781±111 270	66 933±387 480
Total	338	175 434±123 305	38 711±498 440

Note: In aged and young groups, no significant difference was observed in rhGH treatment groups compared the control.
